# Who Serves the Poor? An Equity Analysis of Public and Private Providers of Family Planning and Child Health Services in Kenya

**DOI:** 10.3389/fpubh.2018.00374

**Published:** 2019-01-08

**Authors:** Nirali M. Chakraborty, Dominic Montagu, Joyce Wanderi, Christine Oduor

**Affiliations:** ^1^Metrics for Management, Oakland, CA, United States; ^2^Department of Epidemiology and Biostatistics, University of California, San Francisco, San Francisco, CA, United States; ^3^Population Services Kenya, Nairobi, Kenya

**Keywords:** Kenya, equity, family planning (FP), private sector, child health

## Abstract

Understanding differences in the wealth status of patients can inform planning decisions aimed at providing affordable access to high quality care to all. This study assesses differences in the wealth status of clients of family planning and child health services by health sector. It also describes reason for facility choice, cost of services, and the proportion of additional clients of these services, and assesses if there are any differences by health sector.

A cross-sectional survey of 2,173 clients from 96 health facilities in urban areas of 6 counties in Kenya was conducted, stratified by health facility type. The 4 strata were public, faith-based, private for profit, and social franchise. Client wealth was benchmarked to the national and urban population of the 2014 Kenya Demographic and Health Survey (DHS), and assessed using the EquityTool.

There were significant differences in the client wealth distribution between facility types, and public sector facilities served a significantly higher proportion of poor clients than other types of facilities. In all three non-public facility types, more than 25% of clients were from the poorest two wealth quintiles, without significant differences between facility types. No facility type stands out as expanding access to health services more than another.

Results show that social franchises do better at reaching the poor than earlier studies have indicated, though not as well as faith-based and public facilities. Findings suggest that private providers remain important within the larger health system, more so for family planning than childhood illness management. In urban areas with significant facility choice, this study quantifies differences in client wealth across four health sectors. Incorporating these findings into policy and programmatic interventions can improve equity in access to and use of quality health services.

## Introduction

Understanding where women and children seek care is an important first step in being able to provide affordable access to all and to ensure that the care being received is of high quality (1, 2). Such knowledge can inform supply side interventions addressing availability and affordability, as well as demand side interventions addressing ability to seek, reach and pay for care, ultimately informing program planning (3).

A number of health financing and health system interventions to improve coverage of basic health services have been implemented throughout low- and middle-income countries. These include removal or reduction of user fees in the public sector, targeted vouchers for poor clients, contracting out to the private sector, results-based financing initiatives to incentivize improving coverage of particular health services, quality improvement initiatives, and social franchising of private sector providers. Social franchising is an intervention which organizes independent private sector providers under a common brand, provides quality assurance and training, and demand generation services by the franchisor.

As social franchise organizations and other not-for-profit and for-profit service providers have begun to systematically assess equity by capturing the wealth profile of their clients, questions are emerging regarding what an optimal client wealth distribution should be, and how clients at one facility type compare to another facility type including public, private for profit, and not-for-profit institutions.

The Demographic and Health Surveys (DHS) provide an important source of information on the use of basic health services, with the ability to analyze data by various indicators of socio-economic status. These nationally representative surveys are conducted using a standard base questionnaire, and have occurred in over 90 countries, supported by the United States Agency for International Development (USAID). Surveys are often conducted every 5 years in collaboration with national government agencies. In 2004, Gwatkin and colleagues noted that the use of health services is regressive (with the rich using more services than the poor), even for basic and preventative health services such as immunization, medical treatment of childhood illnesses (fever, diarrhea and respiratory infection), and antenatal care (4). A recent multi-country analysis of DHS data also quantified the extent of socio-economic differences in use of health services by sector and country, generally showing that the public sector provides more services to those in the poorest wealth quintile than the private sector, with the poor more likely to forego care for common childhood illness (5). Yet, the private sector is much more heterogeneous than the public sector, comprising both formal and informal sources of care. Formal sources include clinics and pharmacies, while informal sources include drug shops and community-based distributors. The DHS do not allow for more refined distinctions between types of private sector facilities, differentiation between sizes, patient volume, locale, or whether or not private providers are affiliated with franchised networks or other system of support or quality assurance. DHS are not designed to provide sufficient estimates from each type of facility to be able to compare client characteristics, eliminating nuance between types of private sector facilities and detailed information on equity in service use. Thus, while the information provides a national perspective on use, DHS data cannot tell us what the mix of patients is at any one facility type.

### Context

Kenya, a lower-middle income country undergoing an epidemiologic transition, continues to have wealth-related disparities in use of and access to preventive and curative primary health services as well as differences in quality by service type (6–9) In Kenya, 53% of married women use modern methods of family planning (FP); however, there is significant difference in use between those in the wealthiest and poorest quintiles (57.7 vs. 29.2%) (10). The public sector provides 59.9% of all contraception, the remaining 40% is delivered by retail outlets, NGOs, faith-based organizations, social franchised providers, and for-profit clinics. Faith-based organizations are those run by or overseen by a religious entity.

In the 2 weeks preceding the most recent 2014 DHS survey, 8.5% of children had symptoms of acute respiratory infection (ARI), 24.4% had fever and 15.2% had diarrhea. For all illnesses, care was sought from a health facility or provider for more than 55% of cases, irrespective of wealth quintile (10). Source of treatment varies across wealth quintiles for FP and child health illnesses (5).

The private sector, and in particular social franchising, is well-developed in Kenya, and includes PSKenya's Tunza network and Marie Stopes' Amua network. 2014 surveys by Amua showed that 86% of their clients were from the top two wealth quintiles, when benchmarked against the 2008 Kenya DHS survey (KDHS) (11). Given that overall wealth in Kenya has likely increased between 2008 and 2014, these data may not accurately reflect the socio-economic status of franchised clients. It may also be that in the catchment areas served by social franchises, only the wealthy seek care, and public, faith-based, or private for-profit providers would all have equivalent client profiles. We cannot currently make these comparisons because similar data for other providers is not available.

The primary objective for this study is to assess whether there are differences in the wealth status of clients of FP and child health services by health sector. A secondary objective of the study is to describe the proportion of additional clients of FP and child health services, and assess if there are any differences by health sector. Understanding how sectors differ in proportion of additional users served indicates whether any one sector is superior in improving access to care for these services.

## Methods

### Study Design and Sampling Strategy

An observational, cross-sectional design was employed for this study. The study was restricted to facilities in urban and peri-urban regions only, due to the higher concentration of private sector facilities in these areas. One county from each region except the Nairobi and North-Eastern Regions was selected. These two regions were excluded due to the highly skewed nature of their wealth distributions. The 2014 KDHS indicates that wealth in the other six regions is more equally distributed (10).

Counties without any franchised (Tunza or Amua) or faith based (FBO) facilities were excluded. One county from each region with the highest number of franchised facilities was purposively selected. Within the county, franchised facilities were listed and sorted by sub-county. One sub-county from each county with at least three franchised facilities and three FBO facilities was randomly selected, and a second was randomly selected as the back-up. The back-up was selected in case the facilities in the chosen sub-county did not meet inclusion criteria listed below, or an insufficient number agreed to participate in the study.

### Facility Inclusion Criteria

Facilities were identified from the lists provided by Tunza and Amua franchise managers, as well as the Kenya Master Facility List (MFL), providing details of facility by county, sub-county, facility type, and number of beds. All identified facilities were visited prior to data collection to gain permission to conduct the study, and to assess the facility's suitability for inclusion. Facility in-charge personnel were asked to answer a short questionnaire detailing the types of services offered, and the average number of FP and child health clients seen daily.

Facilities had to be uniquely identified as belonging to one of four categories: 1- public sector (government) health centers or dispensaries; 2- faith based, church, mission hospitals or clinics (FBO); 3- private for-profit hospitals or clinics; 4- private franchised facilities branded as Tunza or Amua. All facilities were located in urban or peri-urban areas, as defined by presence of a daily market and identifiable market center, and reported providing FP and child health services. Level 4 and Level 5 facilities (equivalent to a district or referral hospital) were excluded from the initial frame, because the focus of the study was on primary care services. Having more than 10 inpatient beds was originally included as an exclusion criteria, but not retained due to unreliable data on this factor from the MFL, although efforts were made to select non-hospital private facilities. Facilities that reported seeing on average less than one client per day were excluded.

### Facility Selection

Where possible, facilities were randomly selected after establishing a minimum daily volume of eligible clients required for each facility in that stratum. These minimum daily volumes ranged from three to six clients, depending on the facility type, and determined by the client flow recorded during the eligibility visits.

### Client Inclusion Criteria

Eligible respondents were women aged 18–49 who received a FP service, or guardians of children aged 0–5 who sought care for fever, cough/respiratory infection, or diarrhea (child health services).

### Sample Size

Sample size was calculated to estimate the proportion of clients seeking FP or child health services who are in the poorest 40% of the national population, stratified by facility type, with a desired precision of 5%, power of 80%, and at alpha = 0.05. Data from the 2014 Kenya DHS survey indicating the proportion of users in the bottom 2 wealth quintiles seeking FP services from the public, NGO, and private sector were used to estimate the proportion of users (P) and the maximum sample size require for each stratum. A design effect estimate of 1.8—more conservative than the highest value provided by KDHS 2014 for each sub-population—was applied to the resulting sample sizes. This sample was increased by 10% per stratum for non-response.

### Data Collection

Data collection occurred over 4 weeks in August and September 2016. Potential respondents were screened after services had been sought. Interviewers were instructed to approach any woman appearing to be in the eligible age range, as well as anyone exiting with a child who could be five or younger. If they met the inclusion criteria, they were asked to participate in the survey. There was no quota established for each client type. For each facility, interviewers were given a skip pattern to follow when approaching potentially eligible clients for consent in order to ensure clients representative of all times of the day were interviewed. The skip pattern was derived from estimates of daily client volume and a desire to interview at a given facility for at least 2 days. Consequently, at higher volume facilities, interviewers may have been instructed to skip several potentially eligible clients before approaching the next client, whereas in lower volume facilities they may have been instructed to approach several clients and then skip one client.

### Ethical Approval

Ethical review for this study was received from AMREF (ESRC P247/2016) and the Marie Stopes Ethical Review Board (016-16). Approval to conduct the study was also sought and received from the Kenya Ministry of Health, the Christian Health Association of Kenya, Marie Stopes Kenya, and Population Services Kenya.

### Analysis

Analyses described below are descriptive and were conducted using Stata13. The wealth index results were calculated using the Kenya EquityTool methodology, comparing respondents to both the national and urban wealth distributions (12). This methodology reduces the number of questions that need to be asked to assess relative wealth, and divides the Kenyan population into five quintiles of 20% each, defined by the most recent, 2014, DHS, which is nationally representative (10, 13). Using such a measure ensures that these data are comparable to DHS surveys, and that differences in service utilization can be more easily compared across countries. An additional user was defined as someone who is an initiator of modern contraception (either someone who has never used modern contraception before or someone returning to modern contraceptive use) for FP, and—for child health services—as someone who has never used the formal health sector before for the illness in question, either because they did not previously have a need or because they sought care from an informal source. Variables were assessed for normality graphically, using quantile-normal plots. One outlier observation was removed in the analysis of cost of services. *T*-tests and ANOVA were used to compare continuous variables between and across the four strata, while chi-squared tests were used to compare results for wealth quintile and other categorical variables from the four strata. Results were adjusted for clustering by facility where noted, using the **svy** functions in Stata. Given the multiple comparisons (between each pair within the four strata) in the main study outcomes, statistical significance is determined only after applying the Holm-Bonferroni correction (14).

## Results

### Facility and Respondent Characteristics

Of 248 facilities that were visited, 209 were eligible for study inclusion. County level permission was required to visit public sector facilities, and was obtained without concern in 5 of 6 counties. In Mombasa county, numerous attempts to receive permission were unsuccessful. Given that two ethical review boards and the National Ministry of Health had already reviewed the proposed sample and given permission to conduct the study in the 6 selected counties, it was not considered an option to replace Mombasa county with another county. The allocated public sample from Mombasa was reallocated to the other 5 counties. The FBO sample in Mombasa also had to be reallocated because there were no eligible facilities in the primary or back-up sub-counties. In the other counties, four facilities revoked their permission to conduct the study after the initial visit, and the allocated sample for those facilities was also reallocated to facilities in the same stratum and county, if a replacement facility could not be found. Among those eligible, 96 facilities were chosen to be a part of the study, distributed across 6 counties and four strata.

Three thousand four hundred and fifty-nine individuals were approached to participate in the study, and 3,407 (98.5%) agreed to participate. 63.8% of those who consented to participate were eligible for the study. A total of 2,173 individuals were interviewed, across 96 chosen facilities. Table 1 illustrates the distribution of study facilities and respondents by county and stratum.

**Table 1:** Distribution of facilities by county and stratum.

**County**	**Stratum**	**Facilities**	**Number of respondents interviewed per facility**			
			**Minimum**	**Median**	**Maximum**	**Total**
Nakuru	Public	6	22	24	24	141
	FBO	6	11	30	31	160
	Private	4	13	23	24	83
	Franchise	5	12	20	20	92
Mombasa	Public	0	–	–	–	0
	FBO	0	–	–	–	0
	Private	5	0	19	21	71
	Franchise	6	4	19	21	98
Machakos	Public	5	26	30	31	145
	FBO	3	16	30	42	88
	Private	4	1	19	25	64
	Franchise	6	0	18	28	88
Kakamega	Public	5	10	27	28	103
	FBO	2	6	15	24	30
	Private	4	15	22	26	85
	Franchise	5	2	19	20	79
Homa Bay	Public	5	28	29	33	149
	FBO	3	38	39	42	119
	Private	3	31	33	34	98
	Franchise	4	21	25	25	95
Kiambu	Public	4	29	29	30	117
	FBO	3	10	39	40	84
	Private	4	0	33	52	101
	Franchise	4	10	25	25	83
Total	Public	25	10	28	22	655
	FBO	17	6	30	42	481
	Private	24	0	23	52	502
	Franchise	30	0	20	28	535

**Figure 1:**
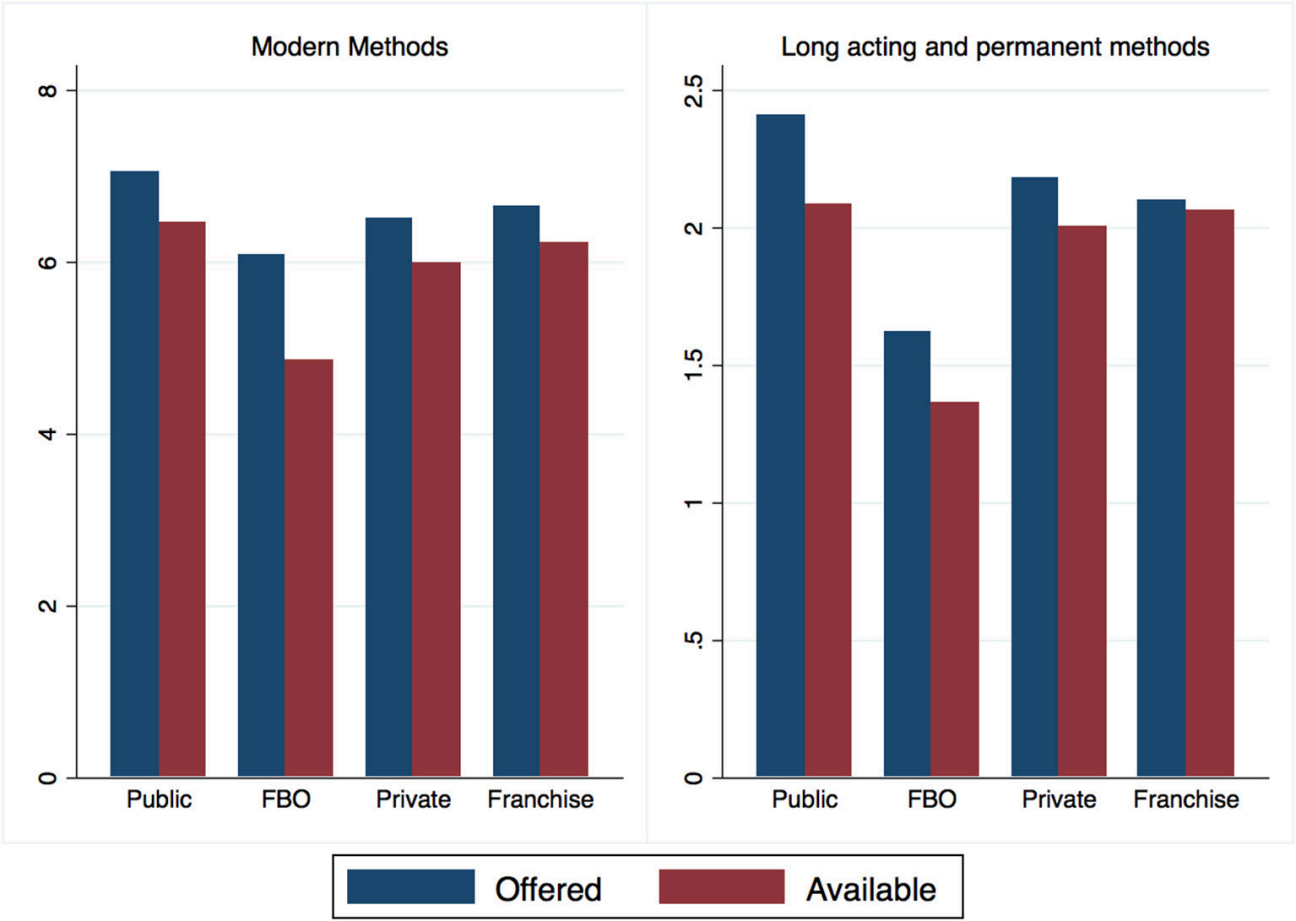
Mean number of FP methods offered and available by facility type.

All facilities were required to provide FP for inclusion in the study; however, there were significant differences (*F* = 4.11, *p* < 0.01) in the number of FP methods available, and the number of long acting and permanent methods (LAPM) available (*F* = 7.57, *p* < 0.001), across facility types. A facility was defined as offering a method if the respondent to the facility stated that the method was offered at that facility. A second question asked if that method was available on the day of the survey. Methods may not be available either due to stock-out or due to lack of trained personnel to provide the method. Public facilities offered and had available the highest number of methods, followed closely by franchised private facilities. While public facilities nominally offered the highest number of LAPM, private franchise facilities had the highest number of LAPM available at the time of reporting (Figure 1). There was also considerable variation among facility sale of medicines and acceptance of insurance. Public facilities were the least likely to sell medicines, while faith based facilities were the most likely.

There were 919 FP clients and 1,254 child health clients. Table 2 illustrates the characteristics of study participants by reason for visit (including stratum and county). The relationship between reason for visit and marital status ($\chi_{3}^{2}=$10.97, *p* = 0.012), and education ($\chi_{2}^{2}=7.53$, *p* = 0.023) were both significantly different. Since no quota for the number of FP clients per facility or stratum was included, comparing the proportion of FP and child health clients in each stratum indicates if the facility type is more likely to see clients for a particular reason. The row percentages in the last panel of table 2 were tested to see if they were significantly different from 50%. In private and franchised clinics, there was no significant difference in the proportion of clients interviewed, by reason for visit. Not shown in Table 2, the vast majority (88%) of both FP and child health clients had been to the facility they visited before, with no significant differences between groups. Clients were also asked why they chose the health facility, and were able to give multiple answers. Common answers were that the facility was nearby (44.4%), had good quality (40.1%), had the services needed (39.8%), was convenient (24.5%) or that providers were nice/friendly (23.6%).

**Table 2:** Client Characteristics by reason for visit.

	**Reason for visit**				**Total (*****n*** **= 2,173)**	
	**FP (*****n*** **= 919)**		**IMCI (*****n*** **= 1254)**		
**Client characteristics**	***n***	**%**	***n***	**%**	***n***	**%**
**GENDER OF RESPONDENT**						
Male	0	0.0	80	6.4	80	3.7
Female	919	100.0	1174	93.6	2093	96.3
MARITAL STATUS^*^						
Married/Living together	758	82.7	1,075	85.9	1,833	84.6
Never married	125	13.6	144	11.5	269	12.4
Divorced/Separated	30	3.3	20	1.6	50	2.3
Widowed	4	0.4	12	1.0	16	0.7
EDUCATION^*^						
No Education	24	2.6	15	1.2	39	1.8
Primary School	351	38.2	451	36.2	802	37.1
Secondary School and Higher	542	59.1	780	62.6	1,322	61.1
**COUNTY**						
Nakuru	214	23.3	262	21	476	21.9
Mombasa	93	10.1	76	6	169	7.8
Machakos	162	17.6	223	18	385	17.7
Kakamega	111	12.1	186	15	297	13.7
Homa Bay	183	19.9	278	22	461	21.2
Kiambu	156	17.0	229	18	385	17.7
**FACILITY TYPE (ROW PERCENT, *P*-VALUE^a^)**						
Public^**^	219	33.4	436	66.6	655	*p* < 0.01
FBO^*^	190	39.5	291	60.5	481	*p* = 0.04
Private	256	51.0	246	49.0	502	*p* = 0.84
Franchise	254	47.5	281	52.5	535	*p* = 0.51
Total^**^		42.3		57.7		*p* < 0.01

a *Significance testing accounts for survey design*.

* *p < 0.05*,

** *p < 0.01*.

Clients were asked how much they paid for services, and if they had health insurance. Nearly 30% of clients had health insurance, but only 4.7% of them used the insurance for the visit. Client payment was assessed for differences between facility type, and difference between reason for visit. There was a significant difference in mean amount paid between the public sector and the other three sectors (*t* ≥ 4.73, *p* < 0.001 for each), but no significant difference between clients of FBO, private, and franchised facilities (Table 3). The finding is the same when comparing mean cost of FP or child health services alone, by sector; however, those seeking care for childhood illnesses spent significantly more than those seeking FP, overall (KSH 263.7 vs. 151.5; *F* = 15.24, *p* < 0.001).

**Table 3:** Amount Paid for Services (in Kenyan Shillings), by Sector Visited or reason for visit.

**Sector**	**Mean**	**25th percentile**	**50th percentile**	**75th percentile**
Public	22.7	0	0	0
FBO	279.3	100	150	350
Private	304.8	100	150	350
Franchise	312.4	100	150	400
FP	151.5	0	100	150
IMCI	263.7	0	100	350

**Figure 2:**
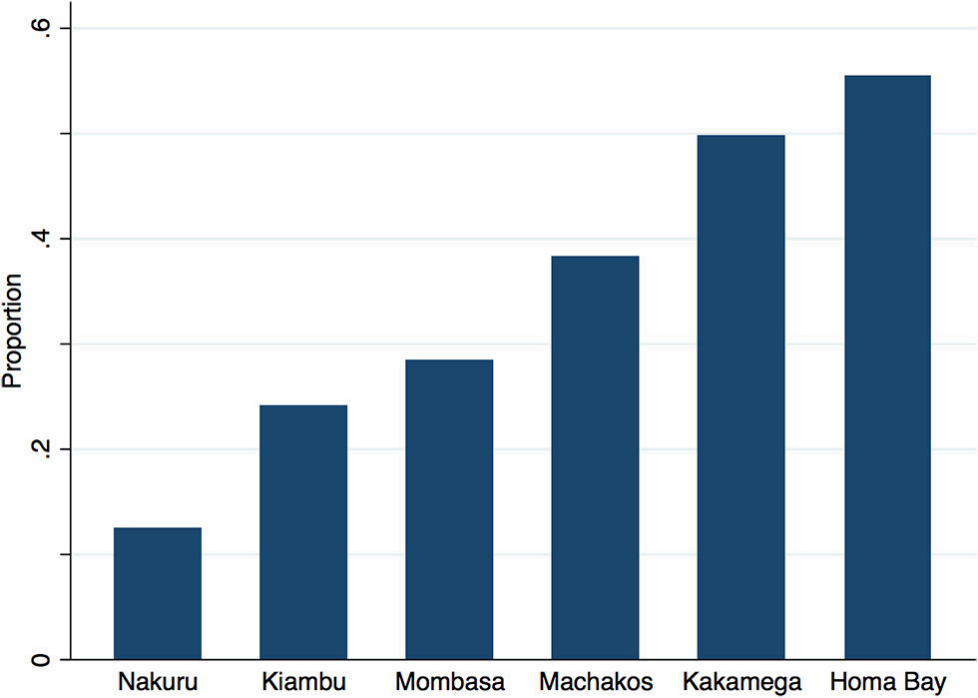
Proportion of clients in lowest two urban quintiles, by county.

Table 4 outlines the overall wealth distribution of study respondents in comparison to national and urban benchmarks. Although clients are distributed evenly across urban wealth quintiles—used as a default benchmark as the facilities surveyed were urban or per-urban—there are marked regional differences in wealth. Approximately 12% of clients across all 4 sectors in Nakuru are in the poorest 2 urban quintiles vs. 50% of clients in Kakamega and 55% of clients in Homa Bay (Figure 2).

**Table 4:** Distribution of Respondents by National and Urban Wealth Quintile (n = 2157)^a^.

**Quintile**	**% in national quintile**	**% in urban quintile**
1 - Poorest	5.7	19.52
2	6.49	15.11
3	11.31	19.1
4	27.07	22.86
5 - Wealthiest	49.42	23.41
Total	100	100

a *16 respondents did not have complete data to calculate their wealth quintile*.

### Client Wealth Distribution by Facility Type

Table 5 shows the distribution of clients by national wealth quintile and facility type. The wealth distribution of clients is significantly different across all four sectors (*F* = 4.13, *p* < 0.001). In pairwise comparisons, wealth distribution between public and private (*F* = 11.82, *p* < 0.001) and public and franchise (*F* = 8.89, *p* < 0.001) sectors are significantly different, using a design-based chi squared test, adjusted for multiple comparisons.

**Table 5:** Proportion of clients in each national wealth quintile, by Sector.

	**Type of facility**				
**National quintile**	**Public (%)**	**FBO (%)**	**Private for-profit (%)**	**Franchise (%)**	**Total (%)**
1 (poorest)	10.92	5.70	1.20	3.55	5.70
2	11.54	6.54	2.41	4.11	6.49
3	16.46	10.34	8.43	8.60	11.31
4	30.92	27.64	26.71	22.24	27.07
5 (wealthiest)	30.15	49.79	61.24	61.50	49.42
Total	100.00	100.00	100.00	100.00	100.00

There is a significant difference in the proportion of clients in the bottom two quintiles of the national distribution (those who are in the poorest 40% of the Kenyan population) across all sectors ($\chi_{3}^{2}=108,\;$*p* < 0.001). There are also significant pairwise differences between the public and private sectors (*t* = 3.88, *p* < 0.001) and between the public and franchise sectors (*t* = 2.83, *p* < 0.01), accounting for survey design and multiple comparisons. The franchise sector serves more of the poorest than the private for-profit sector, but less than the FBO sector, although differences are not statistically significant.

As all facilities surveyed are in urban or peri-urban settings, a more appropriate benchmark may be that shown in Table 6, comparing clients only to urban Kenyan households by facility type. There is a significant difference in the urban wealth distribution of clients across all sectors (*F* = 4.56, *p* < 0.001), and between public and private (*F* = 11.9, *p* < 0.001) and public and franchised (*F* = 11.79, *p* < 0.001) facilities, using a design based chi squared test adjusted for multiple comparisons. The difference between public and faith-based facilities is marginally significant (*p* = 0.057), and no longer so when adjusted. When comparing the proportion of clients in the poorest 40% of the urban wealth distribution, the public sector serves significantly more of the poorest than the private (*t* = 4.25, *p* < 0.001) and franchised (*t* = 4.08, *p* < 0.001) sectors, adjusted for multiple comparisons. There is no significant difference between the proportion of poorest urban residents served between the faith based, franchised and private sectors.

**Table 6:** Proportion of Clients in Each Urban Wealth Quintile, by Sector.

	**Type of facility**				
**Urban quintile**	**Public (%)**	**FBO (%)**	**Private for-profit (%)**	**Franchise (%)**	**Total (%)**
1	32.77	18.78	9.64	13.27	19.52
2	20.46	14.77	13.05	10.84	15.11
3	18.77	19.20	19.28	19.25	19.10
4	17.38	25.11	26.10	24.49	22.86
5	10.62	22.15	31.93	32.15	23.41
Total	100.00	100.00	100.00	100.00	100.00

### Additional Clients by Facility Type

Among 916 women who came for FP, 10.3% had never used a modern method before, and overall, 15.6% of women were additional users of FP (Table 7). There was no significant difference by facility type.

**Table 7:** Proportion of Additional Users of FP, by Sector.

	**Type of facility**				
**Additional FP user**	**Public (%)**	**FBO(%)**	**Private for-profit (%)**	**Franchise (%)**	**Total (%)**
No	84.47	86.32	82.61	84.65	84.39
Yes	15.53	13.68	17.39	15.35	15.61
Total	100.00	100.00	100.00	100.00	100.00

Among child health clients, approximately 25% had never needed a health service before for that issue, with an additional 2.7% of children having been taken to an unqualified provider or no provider for a previous episode of the illness. There were no significant differences in the proportion of additional users across the four facility types (Table 8).

**Table 8:** Proportion of Additional Child Health Clients, by Sector.

	**Type of facility**				
**Additional child health client**	**Public (%)**	**FBO (%)**	**Private for-profit (%)**	**Franchise (%)**	**Total (%)**
No	76.28	67.96	73.06	73.38	73.08
Yes	23.72	32.04	26.94	26.62	26.92
Total	100.00	100.00	100.00	100.00	100.00

## Discussion

This study seeks to understand differences in the patient composition between health service facilities in the public, private, franchised, and faith-based sectors, in urban and peri-urban Kenya, and implicitly to understand if the poor are seeking FP and child health care, and if so from where.

When compared to the national wealth distribution, respondents are predominantly found in the 4th and 5th wealth quintiles overall (Table 4); however, the fairly equitable distribution across urban quintiles supports the hypothesis that clients in the facilities, all urban and peri-urban, themselves also came from urban areas, and that the facility-based sample was not, in aggregate, overly skewed toward either the wealthy or poor.

Public sector facilities were found to serve a higher proportion of poor clients than other types of facilities. These findings may be explained in part by the lower price paid for services in the public sector, where over 75% of clients did not pay anything out of pocket. All three non-public facility types served a cross-section of the urban population, but with a leaning toward wealthier patients. The difference in the proportion of poor clients served by the franchise and for-profit private sectors was not meaningful, and both sectors served fewer poor clients than the faith-based sector, although differences were not statistically significant. Assessment of statistical significance in this study is highly influenced by the clustering of clients within facilities and the larger standard errors which result from this design.

No facility type stands out as expanding access to health services more than another, as measured by the two assessments of additional users (for FP and child health services). Understanding the value of a health facility (or facility type) in expanding access may be more meaningful in rural areas, where facilities are further away from each other. In urban areas, such as those where this study took place, all facility types contribute to expanding access.

It is worthwhile reflecting on the unequal distribution of urban poor health care seekers across the six study sites (Figure 2). The overall distribution of wealth in our study is very similar to the distribution of wealth among urban dwellers of Kenya, yet the poor are not evenly distributed across urban areas. Additionally, some of the urban areas selected for the study may not be representative of all urban areas within that region. For example, author's analysis of the wealth of care-seekers in Nakuru to those of all urban dwellers in the Rift Valley region show distributions which are starkly different (calculated from 2014 KDHS (10)). There are inconsistencies across regions which cannot be fully explained by the argument that care-seekers overall are wealthier than the population from which they are drawn. A more nuanced explanation for the variations we see may be due to barriers in some regions which inhibit the poor from accessing the formal health system. In Nakuru, Machakos, Kakamega, and Homa Bay, care seekers are generally wealthier than the rest of the urban population and the poorest two quintiles are under-represented among care-seekers. In Mombasa, however, care seekers' wealth mirrors the overall urban population, while in Kiambu, care-seekers are poorer than the general urban population. Possible explanations for these two exceptions might be the limited facility-types surveyed (see following paragraph) and more even socio-economic distribution in Mombasa compared to other regions in Kenya; and that in Kiambu, a relatively poor county neighboring Nairobi, the wealthier members of the community seek care in the nearby city, leaving a poorer cross-section of the county to seek care locally.

This study has three principle limitations. First, it captured facilities in only two of four strata in Mombasa, because permission to conduct research in the public sector was not given, and eligible FBO facilities were not found. In order to achieve the full sample, the sample was reallocated, and proportionality of the sample across regions was somewhat compromised. Second, this study is one of the first to use the EquityTool—a validated, shorter version of the DHS wealth index that compares those sampled to the national and urban wealth distributions. The shorter index was chosen due to the benefits conferred when interviewing outside of the household, including a shorter questionnaire and easier questions, but there is some known mis-categorization between adjacent quintiles (13). When interpreting data, it is advisable to look at the overall wealth distribution, rather than focusing on one quintile alone. Third, by design, the inferences drawn from these findings are limited to those who do in fact seek formal health services. The study does not capture those seeking care at drug shops or pharmacies, and the sampling methodology, reliant upon the Kenya MFL and local providers, meant facilities run by unqualified providers, or unregistered facilities were unlikely to be sampled. Nevertheless, this study is representative of services available to nearly 70% of all urban dwellers in Kenya (calculated from 2009 Census of Kenya) (15).

Taken together, the findings suggest that private providers remain important within the larger health system, more so for FP than childhood illness management. In particular, there are a number of findings with implications for social franchises: they do better at reaching the poor than earlier studies have indicated; however, no better than non-franchised private providers, and somewhat worse than FBO and public facilities. Franchises do a good job of making FP accessible, outperformed in this only by the public sector, which provides access but sees proportionally fewer FP clients. While this study indicates that franchises see clients of similar wealth levels as other private providers, recent research in Kenya has shown that franchise providers see a larger number of FP and child health clients (16). Franchises may thus be able to achieve greater impact through volume of poor seen, rather than proportion of poor within any one clinic.

The results indicate that franchised providers in Kenya have had some successes in leveraging private markets to expand the reach and adoption of FP. The target niche, and related social value, of franchised facilities is evident but not strong: franchised facilities serve more, and more additional, FP users than FBO or public sector providers, but numbers are still low in each facility and the poor are not well-targeted. Greater use of demand side financing initiatives could increase access for the relatively poor.

This is the next challenge for social franchises: how to distinguish their role in the health sector either by reaching more poor (through direct or indirect subsidies, greater outreach, or other methods), or by emphasizing quality and access to a degree that franchised providers increase volumes relative to other sources of care, playing a more important role in overall market expansion than they do currently. This study suggests that both options are open to franchise programs, and that a strategy to distinguish franchises from other provider types would be helpful in explaining the value added to the health system by this delivery model. Ultimately, how franchise programs respond to these findings will be determined by the priority they put upon serving the poor or increasing service adopters.

Perhaps surprising are the findings that faith based facilities are not significantly more pro-poor than for-profit private sector facilities (both franchised and independent). These facilities are organized, most often under the Christian Health Association of Kenya, which provides them with access to technical and human resources, and a national level advocacy body. They have a mission to assure equity in access to health care, yet faith based facilities do collect out-of-pocket payments similar to the other two facility types (17). While service cost may play a role in determining health seeking choices among the poor—especially those who would pay nothing for services at public facilities—there were no significant differences in the mean amount paid by clients of non-public facilities (Table 3)—and costs were not reported as important by clients. In fact, women seeking FP services were less likely to visit public sector facilities, despite the lower cost of services and more expansive offering of contraceptive methods. Motivations for selecting providers was not the focus of this study, however; some information can be cautiously inferred. For women seeking FP services, reasons for preferring private and FBO facilities may include real or perceived differences in quality, lower waiting times, or improved provider-client interactions, as established by research in Kenya and elsewhere (18, 19). For FP and child health clients alike, access also remains important. The median travel time for care-seekers was 20 min, with those in the poorest two quintiles traveling longer. While the majority of clients interviewed walked to the facility, suitable formal sector care may not be located near the poorest.

Access to and use of health services is a complicated construct, and it is difficult, if not impossible, to assess all aspects of this construct concurrently. Access for all is an underlying factor in attaining the Sustainable Development Goal of Good Health and Well-Being (Goal 3), especially in achieving universal access to sexual and reproductive health services, and an end to preventable child deaths (20). This study provides additional context for how geographically proximate facilities in urban and peri-urban Kenya are serving clients of different wealth profiles. While the public sector is charged with ensuring access throughout the country, private and faith based facilities are often located where a perceived market exists. This study captures use by those who do visit formal facilities, and indicates that the market is segmented. Such a study has not previously been conducted in Kenya, and is of interest for specific health system actors, as well as for those charged with the universal healthcare mandate. Furthermore, this study contributes to the literature one of the first comparisons of client wealth across facility type, including franchises.

## Author Contributions

NC contributed to the protocol, conducted data collection and analysis, and wrote the manuscript. DM, JW, and CO reviewed the protocol, contributed to analysis, and reviewed the manuscript.

### Conflict of Interest Statement

Two co-authors of this study (JW and CO) are employees of Population Services Kenya, the same organization which manages the Tunza social franchise. The remaining authors declare that the research was conducted in the absence of any commercial or financial relationships that could be construed as a potential conflict of interest.
